# Evaluating Transport Layer Security 1.3 Optimization Strategies for 5G Cross-Border Roaming: A Comprehensive Security and Performance Analysis

**DOI:** 10.3390/s25196144

**Published:** 2025-10-04

**Authors:** Jhury Kevin Lastre, Yongho Ko, Hoseok Kwon, Ilsun You

**Affiliations:** Department of Cyber Security, Kookmin University, Seoul 02707, Republic of Korea; lavelliane@kookmin.ac.kr (J.K.L.); koyh0911@kookmin.ac.kr (Y.K.); hoseok1997@kookmin.ac.kr (H.K.)

**Keywords:** 5G roaming, TLS 1.3, Security Edge Protection Proxy, N32 interface, formal verification, ProVerif, inter-PLMN communication

## Abstract

Cross-border Fifth Generation Mobile Communication (5G) roaming requires secure N32 connections between network operators via Security Edge Protection Proxy (SEPP) interfaces, but current Transport Layer Security (TLS) 1.3 implementations face a critical trade-off between connection latency and security guarantees. Standard TLS 1.3 optimization modes either compromise Perfect Forward Secrecy (PFS) or suffer from replay vulnerabilities, while full handshakes impose excessive latency penalties for time-sensitive roaming services. This research introduces Zero Round Trip Time Forward Secrecy (0-RTT FS), a novel protocol extension that achieves zero round-trip performance while maintaining comprehensive security properties, including PFS and replay protection. Our solution addresses the fundamental limitation where existing TLS 1.3 optimizations sacrifice security for performance in international roaming scenarios. Through formal verification using ProVerif and comprehensive performance evaluation, we demonstrate that 0-RTT FS delivers 195.0 μs handshake latency (only 17% overhead compared to insecure 0-RTT) while providing full security guarantees that standard modes cannot achieve. Security analysis reveals critical replay vulnerabilities in all existing standard TLS 1.3 optimization modes, which our proposed approach successfully mitigates. The research provides operators with a decision framework for configuring sub-millisecond secure handshakes in next-generation roaming services, enabling both optimal performance and robust security for global 5G connectivity.

## 1. Introduction

Fifth Generation Mobile Communication (5G) technology has established itself as a cornerstone pillar for modern telecommunications, facilitating ultra-high-speed data transfer and ultra-low-latency services that support the growth of globally interconnected applications [1]. As 5G networks expand worldwide, cross-border roaming functionality has become indispensable, serving millions of users who depend on uninterrupted international mobile services for business operations, travel requirements, and everyday communication needs. The foundation of this international connectivity relies on the N32 interface operating between Security Edge Protection Proxies (SEPPs), which ensures secure communication across Public Land Mobile Networks (PLMNs) [2].

The N32 interface operates through a two-phase framework designed to establish and maintain secure communications between network functions. The initial N32-c phase handles system establishment by negotiating capabilities and configuring security parameters, with 3rd Generation Partnership Project (3GPP) standards mandating the use of Transport Layer Security (TLS) [3] 1.3 full handshake protocols for primary authentication [2,4]. Once established, the N32-f phase takes over to manage ongoing service message transmission, offering flexible communication pathways—either through direct bilateral exchanges using TLS 1.3 full handshake mechanisms, or through mediated scenarios that implement the Protocol for N32 Interconnect Security (PRINS) framework [2,4], while the full handshake implementation is mandatory according to 3GPP standards, TLS 1.3 itself inherently provides optimization capabilities, including Pre-Shared Key (PSK), PSK-(Elliptic Curve) Diffie–Hellman Ephemeral (PSK-(EC)DHE), and Zero Round Trip Time (0-RTT) modes, which enable efficient session resumption that reduces computational overhead and enhances overall network performance.

A critical challenge in current N32-f implementations is the mandatory use of the TLS 1.3 full handshake for every connection, which incurs substantial latency overhead and degrades user experience in international roaming. This issue is amplified by the inherent round-trip delays of cross-border communications, where authentication often traverses multiple network hops across continents. Although TLS 1.3 provides optimization modes such as PSK-based resumption and 0-RTT that could significantly reduce connection setup time, their applicability and security implications for N32-f remain unexamined. Addressing this gap is crucial, as mobile services demand both ultra-low latency and rigorous security. To date, no work has offered a comprehensive, evidence-based assessment that combines formal verification and controlled performance evaluation of TLS 1.3 optimization strategies in the context of 5G roaming. Such analysis is particularly urgent given that N32-based inter-operator authentication must span multiple administrative domains, endure variable latency conditions, and still satisfy strict international security requirements [5].

To date, no prior work has provided a comprehensive, evidence-based assessment that integrates formal security verification with controlled performance evaluation of TLS 1.3 optimization strategies specifically for 5G roaming environments. The absence of such analysis is particularly critical given that inter-operator authentication over N32 involves signaling that traverses multiple administrative domains, experiences variable latency conditions, and must comply with strict international security requirements [5].

Motivated by these challenges, this paper presents the first systematic evaluation of TLS 1.3 handshake options for 5G roaming, combining:

- Comprehensive analysis of the 5G roaming authentication environment, identifying operational constraints, threat vectors, and security requirements specific to cross-border inter-operator communication.
- Formal verification of each TLS 1.3 option’s cryptographic properties—confidentiality, integrity, authentication, and PFS—using *pi*-calculus–based ProVerif, aligned with the highest assurance level defined in ISO/IEC 29128-1:2023 [6].
- Controlled performance analysis of N32-c negotiation and N32-f protection under roaming conditions, measuring handshake latency, and computational overhead.
- Proposal of 0-RTT FS as an option for TLS 1.3 handshake that balances performance and security in the N32 interface for 5G roaming scenarios.
- Proposal of a deployment-oriented decision framework that leverages the identified security–performance trade-offs to guide MNOs in selecting TLS 1.3 modes according to roaming requirements, threat models, and application criticality.

The remainder of this paper is organized as follows: Section 2 reviews related work on TLS 1.3 and 5G roaming security. Section 3 analyzes TLS 1.3 handshake options for the N32 interface and introduces our proposed 0-RTT FS approach. Section 4 presents the formal verification model and methodology using ProVerif. Section 5 describes the simulation environment, experimental setup, and performance results under varied roaming conditions. Section 6 synthesizes the identified security–performance trade-offs into a practical deployment decision framework, and Section 7 concludes with final remarks and future research directions.

## 2. Background and Related Work

### 2.1. 5G Network Architecture and Roaming

5G’s service-based architecture (SBA) fundamentally transforms cross-border roaming through disaggregated network functions and enhanced security mechanisms. The evolution from 4th Generation Mobile Network Technology (4G)/Long Term Evolution (LTE) elements to 5G equivalents creates new optimization opportunities: Access and Mobility Management Function (AMF) replaces the Mobility Management Entity (MME) with enhanced mobility management, Session Management Function (SMF) [7] supersedes the Packet Data Network Gateway Control Plane (PGW-C) with improved session handling, and User Plane Function (UPF) evolution from Packet Data Network Gateway User Plane (PGW-U) enables flexible deployment models crucial for roaming optimization.

The architectural transformation enables two primary roaming models with distinct performance characteristics. Local Breakout (LBO) roaming terminates data traffic locally in the visited network [8], achieving reduced latency for internet-bound services but requiring enhanced security coordination. Home Routed (HR) roaming maintains data paths through the home network, preserving service policies while introducing additional latency overhead [9] that TLS optimizations can potentially mitigate.

**Figure 1:**
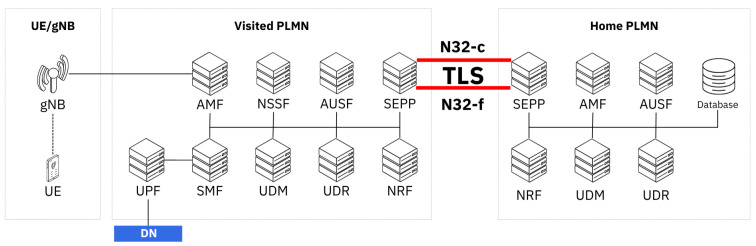
5G core network with a roaming scenario.

The evolution from legacy diameter-based roaming to 5G Service-Based Architecture fundamentally transforms international mobile connectivity through the SEPP framework [10]. Figure 1 shows this architectural shift which introduces the N32 interface as the critical communication pathway between home and visiting networks, operating through two distinct phases: N32-c for initial handshake and capability negotiation, and N32-f for ongoing message forwarding with application-layer security protection.

The SEPP deployment model offers two primary security approaches that directly impact TLS 1.3 implementation strategies. The direct TLS model enables bilateral connections between operators, providing optimal performance through standard TLS 1.3 handshakes. However, this approach requires extensive bilateral certificate management and may not accommodate complex roaming value chains. Alternatively, the Protocol for N32 Interconnect Security (PRINS) model supports mediated roaming through IP eXchange (IPX) providers but introduces substantial performance overhead. PRINS implementation adds 50–100 ms latency compared to direct TLS connections while reducing throughput by 15–25% due to JSON message reformatting requirements and additional cryptographic operations [2]. Although PRINS introduces additional overhead compared to direct TLS channels, many operators still deploy it because it aligns with existing roaming hub and IPX business models. By using PRINS, operators can delegate trust management, routing, and interoperability to centralized intermediaries, which reduces the need for maintaining numerous bilateral TLS agreements with individual partners [11]. In addition, hub providers often offer value-added services such as signaling analytics, filtering, and policy enforcement, which operators may prefer to retain in their roaming ecosystem [12]. These operational and business factors help explain why PRINS continues to see adoption despite its higher performance cost.

Despite these deployment model options, current industry deployments reveal concerning performance challenges that underscore the critical need for TLS 1.3 optimization. The complex certificate management requirements, manual policy negotiations, and limited SEPP vendor ecosystem create substantial operational bottlenecks that impact user experience and deployment timelines.

### 2.2. Advancements in TLS Enable Mobile Network Optimization Opportunities

The TLS 1.3 specification, defined in Request for Comments (RFCs) 8446, introduces fundamental improvements that directly address mobile network performance requirements [3]. The reduction from 2-RTT to 1-RTT handshake completion provides immediate latency benefits, particularly valuable in high-latency mobile environments where RTT can exceed 200 ms. The protocol’s 0-RTT resumption capability enables immediate application data transmission [13] for returning clients, potentially eliminating handshake latency entirely for frequent roaming scenarios.

TLS 1.3 also removes obsolete and vulnerable features from earlier versions, such as static Rivest–Shamir–Adleman (RSA) key exchange and compression. This results in a smaller attack surface and reduces message complexity, which is especially beneficial for mobile devices with constrained processing capabilities [14]. Mobile-specific optimizations include support for compressed certificates, defined in RFC 8879, which significantly reduce handshake size and bandwidth usage. This is crucial for mobile connections that are often metered or limited in throughput [15].

The streamlined cipher suite negotiation process in TLS 1.3 allows for adaptive cipher selection based on device capabilities or network conditions. This supports more efficient cryptographic operations, helping to balance performance and energy consumption. TLS 1.3 mandates forward secrecy and restricts cipher suite choices to AEAD algorithms, such as AES-GCM and ChaCha20-Poly1305. This enhances both security and performance across various hardware platforms, including those that lack cryptographic acceleration [16].

Mobile-specific optimizations include compressed certificate support to reduce bandwidth consumption and adaptive cipher suite selection based on network conditions. Research on 450 MHz LTE-M networks demonstrates successful TLS 1.3 deployment in critical infrastructure applications, showing reduced power consumption and improved reliability in constrained environments [17]. Performance analysis reveals wolfSSL implementations showing 15% improvement in RSA handshakes and 6–7% improvements in (EC)DHE scenarios. The protocol’s enhanced key derivation using HKDF provides more efficient key material generation, particularly beneficial for mobile devices with limited computational resources. Cloudflare reports that 40% of TLS sessions utilize resumption, indicating substantial real-world optimization potential for roaming scenarios.

### 2.3. TLS 1.3 Full Handshake and PSK Option

TLS 1.3’s deployment on the N32 interface is highly efficient for secure roaming communication establishment between SEPPs, which is crucial for the 5G roaming architecture. The ability to establish and resume sessions quickly using various handshake modes minimizes latency and computational overhead, enabling faster roaming setup compared with traditional approaches. This reduction in handshake latency is particularly beneficial in roaming scenarios where efficient and swift inter-Public Land Mobile Network (PLMN) communication is essential. This paper covers the five TLS 1.3 scenarios:

- **Full handshake** provides comprehensive security through complete certificate verification and key exchange. Offers strongest initial security guarantees with PFS, ideal for first-time roaming partner establishments between previously unconnected PLMNs.
- **PSK-Only** relies solely on PSKs for authentication and session establishment. Efficient but lacks PFS. Suitable when PSKs are securely managed and key compromise risk is minimal.
- **PSK-(EC)DHE** combines PSK authentication with ephemeral Diffie–Hellman key exchange. Provides both mutual authentication and PFS, making it suitable for high-security roaming scenarios.
- **0-RTT** enables immediate roaming data transmission in the first handshake message, reducing latency for session resumption. Uses PSKs but has security risks, including replay attacks and limited forward secrecy.
- **0-RTT FS** combines 0-RTT performance with PFS and anti-replay using resumption tickets containing server ephemeral public keys and server generated nonce. Clients perform local ephemeral DH to derive forward-secure early data encryption keys.

The TLS 1.3 options for N32 are suitable for roaming resumption scenarios because they can be secured with operator-negotiated credentials while also providing efficient, low-latency session establishment and resumption, which are essential for the quick and secure interactions required between SEPPs in different PLMNs. These options reduce computational overhead and resource usage, making them ideal for high-volume roaming traffic while maintaining strong security. Additionally, the ability to reuse established sessions simplifies roaming management and aligns well with N32’s need for streamlined and secure inter-operator communication, enhancing the overall performance and security of roaming services.

### 2.4. TLS 1.3 Protocol-Level Vulnerabilities

While TLS 1.3 represents a significant cryptographic advancement over previous versions, formal analysis has revealed several protocol-level vulnerabilities and cryptographic design limitations that affect the security guarantees of the protocol itself. These vulnerabilities stem from fundamental design choices in the protocol specification rather than implementation flaws, highlighting the inherent challenges in designing secure cryptographic protocols.

The most prominent cryptographic vulnerability in TLS 1.3 is the 0-RTT replay attack, which fundamentally compromises the protocol’s security model for early data transmission. However, this optimization introduces critical weaknesses: the early data lacks replay protection and provides only limited forward secrecy [18,19]. In particular, early data keys are derived exclusively from previously established PSKs without fresh (EC)DHE contribution, meaning that compromise of the PSK enables retrospective decryption of all 0-RTT data. Forward secrecy is restored once the handshake completes, but the early data itself remains vulnerable.

Protocol-level downgrade attacks remain viable against TLS 1.3 through cryptographic design limitations in version negotiation. Despite TLS 1.3’s enhanced downgrade protection mechanisms, the protocol’s backward compatibility requirements create cryptographic vulnerabilities that enable sophisticated downgrade attacks [20]. These attacks exploit the cryptographic design of the version negotiation process, where certain combinations of supported cipher suites and protocol versions can be manipulated by attackers to force connections into weaker cryptographic modes. The attacks succeed by exploiting gaps in the cryptographic binding between negotiated parameters and the resulting security context, allowing attackers to manipulate the handshake flow while maintaining valid cryptographic signatures.

Forward secrecy limitations in TLS 1.3’s PSK modes create long-term cryptographic vulnerabilities. Unlike the full TLS 1.3 handshake which provides PFS through ephemeral key exchange, PSK-only modes rely on long-term shared secrets that compromise the cryptographic property of forward secrecy [21]. If the PSK is compromised, all past and future communications using that PSK become cryptographically vulnerable to decryption. This represents a fundamental trade-off in the protocol design where performance optimization through PSK modes comes at the cost of long-term cryptographic security. Even when PSK is combined with ephemeral Diffie–Hellman key exchange, the initial authentication and key derivation steps still depend on the long-term PSK, creating a window of cryptographic vulnerability.

Mixed authentication modes in TLS 1.3 create cryptographic binding issues that can be exploited to bypass security guarantees. When TLS 1.3 combines different authentication mechanisms, such as certificate-based authentication with PSK, the cryptographic binding between these different security contexts may be insufficient [19]. This creates opportunities for attacks that exploit the transitions between different cryptographic modes within a single handshake, potentially allowing attackers to gain stronger authentication guarantees than they should possess or to manipulate the security context in ways that violate the protocol’s intended security model.

Session resumption cryptographic vulnerabilities create ongoing security risks through the reuse of cryptographic material. The protocol’s session resumption mechanism, while providing performance benefits, extends the cryptographic lifetime of key material beyond individual sessions [19]. This creates a fundamental tension between performance optimization and cryptographic security, as longer-lived cryptographic secrets provide larger attack windows and reduce the overall security guarantees of the protocol.

These protocol-level vulnerabilities demonstrate that even carefully designed modern protocols like TLS 1.3 [3] face fundamental security challenges that arise from the inherent complexity of cryptographic protocol design. The extensive formal analysis efforts that have characterized TLS 1.3’s evaluation [18,22,23] continue to reveal subtle but significant vulnerabilities that require ongoing attention from both the cryptographic research community and protocol implementers.

### 2.5. Direct TLS Approach

**Figure 2:**
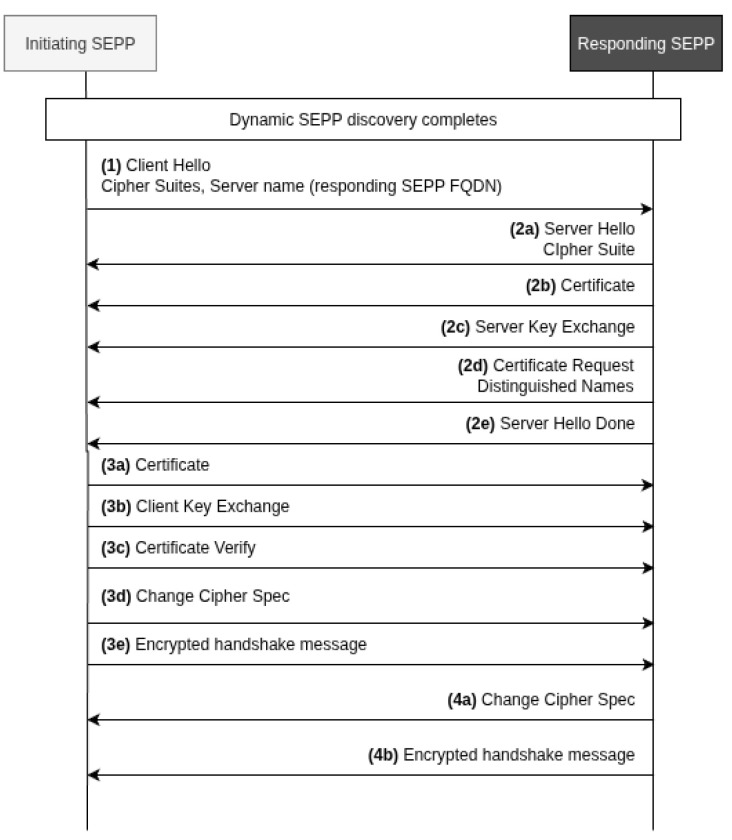
Typical 5G Roaming Direct TLS Setup.

In the Direct TLS model, SEPPs from two distinct PLMNs establish mutual TLS (mTLS) connections without the involvement of intermediary service providers or roaming hubs. The architecture maintains a clear security perimeter where each PLMN retains full control over its cryptographic keys, certificate management, and trust relationships. This bilateral approach ensures that sensitive signaling information traverses only a single encrypted tunnel between the communicating parties, providing the strongest possible security guarantees for inter-PLMN communication. Figure 2 shows the typical roaming TLS connection setup between the initiating SEPP (i-SEPP) and the responding SEPP (r-SEPP). The Direct TLS establishment process involves two distinct phases: the N32-c (control plane) handshake for capability negotiation and security parameter exchange, and the N32-f (forwarding plane) connection setup for actual service message transmission. Both connections utilize separate mTLS tunnels, with the N32-c connection typically being short-lived and used for periodic control exchanges, while N32-f connections are maintained as long-lived channels optimized for high-throughput message forwarding.

**Step 1: Client Hello Initiation.** The initiating SEPP (i-SEPP) establishes a TCP connection to the responding SEPP (r-SEPP) and sends a TLS Client Hello message to initiate the TLS handshake. This message includes the supported cipher suites and adds the selected r-SEPP FQDN(s) to the Server Name Indication (SNI) extension, enabling the r-SEPP to select the appropriate certificate if multiple certificates are available.

**Step 2: Server Certificate Exchange and Key Setup.** The r-SEPP responds according to TLS protocols by sending a sequence of messages: Server Hello with the selected cipher suite (2a), Certificate containing the certificate chain up to but not including the root Certificate Authority (CA) (2b), Server Key Exchange (2c), Certificate Request which may indicate trusted Root CAs (2d), and Server Hello Done (2e). The leaf server certificate contains the r-SEPP FQDN in the Common Name and includes all PLMN IDs in the SAN records for which the SEPP intends to use the N32 connection. The certificate is signed by the subCA certificate of the respective PLMN, which in turn is signed by the root CA of the respective PLMN.

**Step 3: Client Certificate Authentication and Key Exchange.** The i-SEPP performs certificate validation by matching the PLMN ID of the well-known FQDN to the SAN records and verifying the certificate chain against the list of Root CAs in the trust anchor. Subsequently, the i-SEPP sends its TLS client certificate to the r-SEPP containing its FQDN in the CN and SAN records with additional FQDNs for each supported PLMN ID (3a), followed by Client Key Exchange (3b), Certificate Verify with the certificate verification result (3c), Change Cipher Spec (3d), and Encrypted Handshake Message (3e).

**Step 4: Handshake Completion and Secure Channel Establishment.** The r-SEPP performs the same security checks as the i-SEPP, verifying that at least one SAN entry in the client TLS certificate contains a PLMN ID and that the certificate chain is anchored at one of the root CAs included in the selected trust anchor. The handshake concludes with the r-SEPP sending Change Cipher Spec (4a) and Encrypted Handshake Message (4b), completing the bidirectional mTLS handshake and establishing the secure communication channel between both PLMNs.

#### 2.5.1. N32-c Handshake

**Figure 3:**
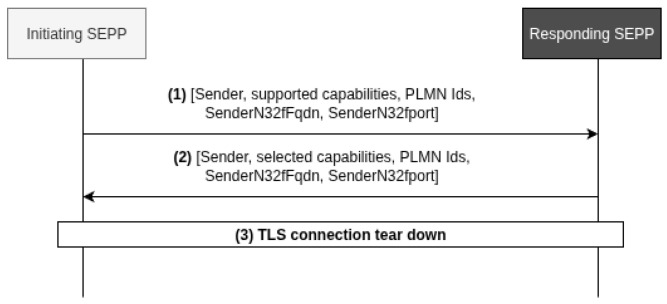
N32-c handshake.

The N32-c handshake procedure serves to create an N32-f context, which represents a communication session between two network endpoints identified by their FQDN:PORT combinations. This N32-f context is defined as a bidirectional connection pair linking the FQDN:PORT of the initiating SEPP with the FQDN:PORT of the responding SEPP. Figure 3 shows the N32-c handshake process.

#### 2.5.2. N32-f Connection Setup

Following the completion of the N32-c handshake, a persistent mTLS connection can be established from the initiating SEPP in PLMN A to the responding SEPP in PLMN B to handle Network Function service requests originating from PLMN A and destined for PLMN B. Additionally, a second persistent mTLS connection may be established in the reverse direction, from PLMN B to PLMN A, to accommodate NF service requests flowing from PLMN B back to PLMN A. The same procedure and security checks as defined in Section 2.5 shall be used to set up the long-lived mTLS tunnel from i-SEPP to r-SEPP.

## 3. N32 Interface: TLS 1.3 Handshake Options for Inter-PLMN Communication

### 3.1. Options (a–d): Standard TLS 1.3 Resumption Modes

The N32 interface facilitates secure communication between SEPPs across different PLMNs in 5G architecture. This section analyzes the TLS 1.3 handshake options available in the N32 interface, with emphasis on their role during session resumption and reconnection. In accordance with 3GPP standards and Global System for Mobile Communications Association (GSMA) guidelines, the initial inter-PLMN connection establishment over N32-c and N32-f must always use a full certificate-based mutual TLS (mTLS) handshake anchored in the GSMA Public Key Infrastructure (PKI). Only after such an initial authenticated session has been established can PSK-based resumption options be considered.

The N32 interface implements a two-phase security establishment process: the initial N32-c (N32 control) handshake for capability negotiation, followed by N32-f (N32 forwarding) connection setup for long-lived inter-PLMN data exchange. The initial phase always requires a full mTLS handshake, while subsequent reconnections may employ one of several TLS 1.3 resumption modes:

1. **Option (a)—Certificate-Based full handshake (Initial Setup)** employs X.509 certificate chains with PLMN identifiers embedded in SAN extensions. Provides mutual authentication through ECDSA signatures and achieves forward secrecy via (EC)DHE key exchange. Certificate validation against GSMA/RAEX trust anchors ensures PLMN identity verification. This mode is mandatory for the first connection between SEPP peers.
2. **Option (b)—Resumption: PSK-Only Mode** utilizes a resumption PSK (PSK) derived from a prior full mTLS handshake. No certificate exchange occurs, reducing latency and overhead. However, forward secrecy is not preserved since the session relies solely on the previously derived PSK.
3. **Option (c)—Resumption: PSK with (EC)DHE** combines resumption PSK authentication with ephemeral key exchange, providing both efficiency and forward secrecy. The hybrid approach derives the handshake secret from both the PSK and a fresh (EC)DHE shared secret: Handshake_Secret = HKDF-Extract(Early_Secret, (EC)DHE_shared_secret). This mode is generally recommended for reconnections.
4. **Option (d)—Resumption: PSK with 0-RTT** extends PSK-based resumption by allowing early data transmission, reducing reconnection latency. Early application data are protected using the early_traffic_secret derived during the resumption handshake, while attractive for performance, this mode is generally considered unsafe for SEPP roaming due to replay attack risks, and is disabled in most deployments.

**Figure 4:**
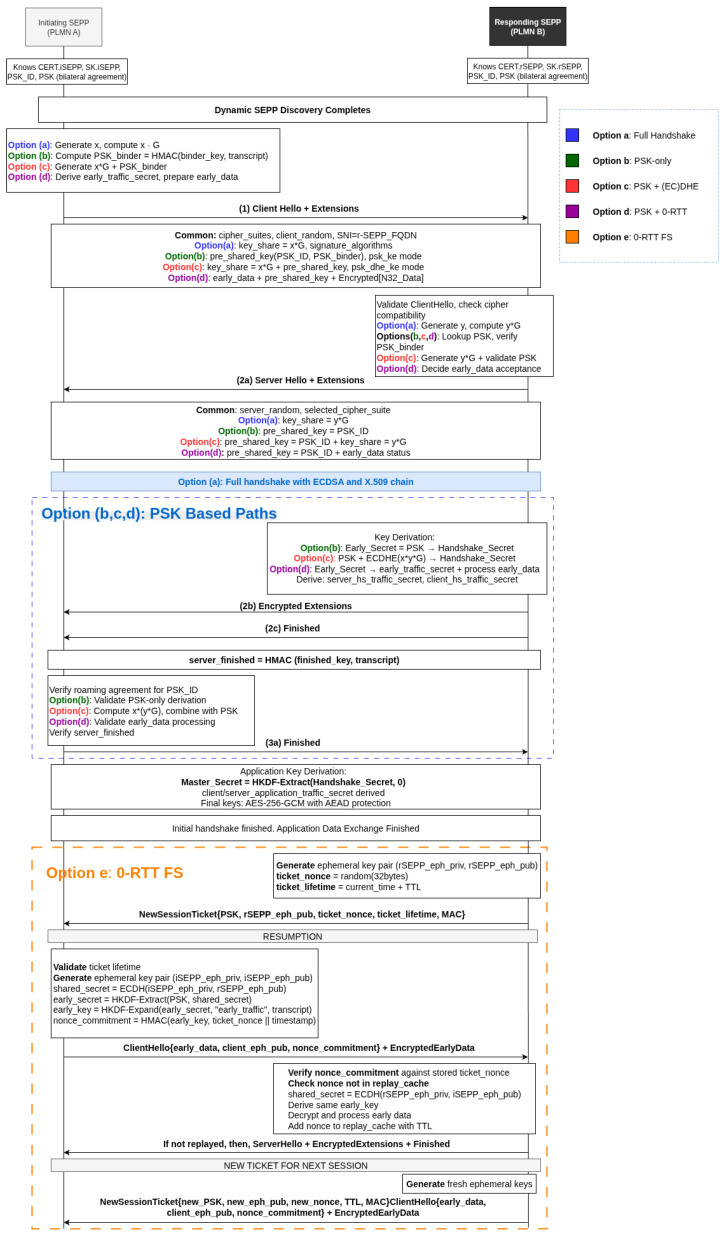
TLS connection procedure showing full mTLS handshake and resumption options.

For N32 security establishment between PLMNs, the initial connection must use a full certificate-based TLS 1.3 handshake with mutual authentication (mTLS). Once this secure session is established, subsequent connections can leverage PSK-based resumption options, including PSK-only, PSK-(EC)DHE, and PSK with 0-RTT, to achieve performance improvements while maintaining varying levels of security assurance. Figure 4 illustrates these TLS 1.3 handshake options as they relate to N32 resumption scenarios, while Section Abbreviations provides definitions for the notations and symbols used throughout this analysis.

### 3.2. The Proposed Option (e): 0-RTT FS with Forward Secrecy and Replay Protection

We propose a new approach called **0-RTT FS**, as **Option (e)** shown in Figure 4, which enhances TLS 1.3’s early data mechanism by adding PFS while maintaining zero round-trip latency. Standard 0-RTT derives early-data keys solely from a PSK, making past communications vulnerable if the PSK is later compromised.

0-RTT FS solves this by combining the PSK with a fresh ephemeral Diffie–Hellman shared secret, computed between a client-generated ephemeral key and a server ephemeral key embedded in the resumption ticket. Each resumption ticket contains both the server’s ephemeral public key and a unique server-generated nonce that are cryptographically bound into the key derivation process. The early-data encryption keys are derived from the combination of PSK, ephemeral DH shared secret, and server nonce using HKDF, ensuring that compromised long-term keys or expired tickets cannot decrypt past early data. The protocol implements anti-replay protection through a simple yet effective mechanism: each server-generated nonce embedded in tickets can only be used once and is tracked in a server-side cache with configurable Time To Live (TTL). Replay attacks are detected and rejected when the server nonce has already been consumed, providing strong replay resistance with minimal server state requirements. The security benefits of forward secrecy and enhanced replay protection justify this trade-off for security-sensitive applications.

All options terminate with the establishment of bidirectional N32-f connections protected by AES-256-GCM with AEAD. The security properties vary by option: certificate-based modes (a) provide PKI-based trust establishment, while PSK modes (b,c,d) rely on bilateral agreements. Forward secrecy is preserved in options (a) and (c) through (EC)DHE key exchange, while option (d) additionally enables 0-RTT data transmission for latency-critical applications. Option (e) uniquely combines the latency advantage of 0-RTT with full forward secrecy and robust replay protection, achieved through ephemeral key integration and single-use server nonce validation.

## 4. Formal Verification

**Figure 5:**
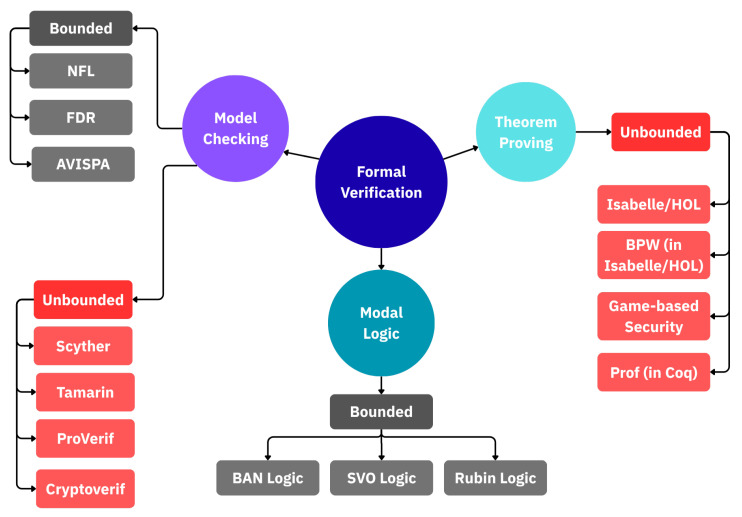
Formal verification tools.

Formal verification is necessary because manual analysis alone often fails to uncover subtle flaws in cryptographic protocols [24,25], and vulnerabilities in handshake design can have critical consequences in 5G roaming security. To ensure rigorous evaluation of TLS 1.3 handshake variants, we employ ProVerif, a symbolic model checker based on the applied pi-calculus and the Dolev–Yao adversary model. ProVerif has been widely used for protocols such as TLS 1.3 and Signal [26,27], and its automated verification of secrecy, authentication, and correspondence properties makes it well-suited for our roaming context. As shown in Figure 5, ProVerif belongs to the category of unbounded model checking tools, meaning it can analyze protocols with an arbitrary number of sessions and messages rather than being restricted to finite state spaces.

### 4.1. Security Models and Algorithmic Framework

The paper implements six key algorithms for formal verification using ProVerif with pi-calculus methodology aligned with ISO/IEC 29128-1:2023 standards.

Algorithm 1 establishes the formal verification framework by defining channel specifications for secure communication, security requirement verification queries (Q1–Q6), and event definitions for protocol step validation. This foundational algorithm creates the symbolic model checker environment under the Dolev–Yao adversary model, where the attacker has complete control over the network but cannot break cryptographic primitives.
**Algorithm 1** Declaration & Queries1:(* Channel specification *)2:free c: channel.3:free sp: channel [private].4:(* Type specification *)5:type G, exponent, label, keyid, key.6:(* Application Data specification *)7:free App Data1, App Data2, App Data3: bitstring [private].8:(* TLS 1.3 Label specification *)9:const p_b_binder, c_e_traffic : label.10:(* Queries specification *)11:event S_STEP1_C_to_S(keyid, key, bitstring, bitstring, bitstring).12:event E_STEP1_C_to_S(keyid, key, bitstring, bitstring, bitstring).13:event S_STEP2_S_to_C(keyid, key, label, bitstring).14:event E_STEP2_S_to_C(keyid, key, label, bitstring).15:event S_STEP3_C_to_S(keyid, key, label, bitstring).16:event E_STEP3_C_to_S(keyid, key, label, bitstring).17:(* Security requirements verification *)18:query kid: keyid, psk: key, rand: bitstring, binder: bitstring, ClienttHello: bitstring;19:**Q1:** inj-event(E_STEP1_C_to_S(kid, psk, rand, binder, ClienttHello))20:    ==> inj-event(S_STEP1_C_to_S(kid, psk, rand, binder, ClienttHello)).21:query kid: keyid, k: key, lb_finished: label, aead_finished: bitstring;22:**Q2:** inj-event(E_STEP2_S_to_C(kid, k, lb_finished, aead_finished))23:    ==> inj-event(S_STEP2_S_to_C(kid, k, lb_finished, aead_finished)).24:query kid: keyid, k: key, lb_finished: label, aead_finished: bitstring;25:**Q3:** inj-event(E_STEP3_C_to_S(kid, k, lb_finished, aead_finished))26:    ==> inj-event(S_STEP3_C_to_S(kid, k, lb_finished, aead_finished)).27:(* Secrecy verification *)28:**Q4:** query attacker(App Data1).29:**Q5:** query attacker(App Data2).30:**Q6:** query attacker(App Data3).

Algorithm 2 initializes a fresh ‘session_id’ and ‘psk’, then runs vSEPP (client) and hSEPP (server) in parallel with unbounded replication to allow arbitrary interleavings and replays. In ‘phase 1’, it outputs ‘initial_psk’ on ‘c’ to model post-handshake key exposure, enabling forward secrecy evaluation.

Algorithm 3 implements the full handshake approach with complete certificate-based authentication, featuring mutual certificate verification against RAEX trust anchors, (EC)DHE key exchange for PFS, and PLMN ID validation in certificate SAN records. This algorithm provides the strongest initial security guarantees through comprehensive certificate verification and key exchange, making it ideal for first-time roaming partner establishments between previously unconnected PLMNs.

Algorithm 4 presents the PSK-only mode utilizing PSK authentication with efficient key derivation without certificate overhead, HKDF-based traffic key generation, and bilateral PSK agreement validation, while this approach eliminates certificate exchange overhead, it sacrifices forward secrecy, making it suitable when PSKs are securely managed and key compromise risk is minimal.

Algorithm 5 introduces the PSK-(EC)DHE hybrid approach that combines PSK efficiency with forward secrecy through PSK authentication with ephemeral key exchange, DHE shared secret integration into handshake secret derivation, and a balanced security-performance approach. This hybrid method provides both mutual authentication and PFS, making it suitable for high-security roaming scenarios.

Algorithm 6 implements standard 0-RTT with early data transmission capability, featuring immediate application data transmission, early traffic secret derivation, and session resumption capabilities. This algorithm reduces handshake latency for time-sensitive 5G network function communications but introduces security risks, including replay attacks and limited forward secrecy.

Algorithm 7 presents the novel 0-RTT FS contribution, enhancing standard 0-RTT with server ephemeral keys embedded in resumption tickets, client ephemeral key generation for each session, and combined PSK+DH secret derivation for forward secrecy. This innovative approach addresses fundamental limitations in standard TLS 1.3 early data mechanisms.

Table 1 summarizes the formal verification queries defined in our ProVerif model. Queries Q1–Q3 verify correspondence and injectivity properties that ensure mutual authentication and secure key exchange between client and server events. Queries Q4–Q6 focus on secrecy properties, checking whether application data remains confidential against an active Dolev–Yao adversary. Together, these six queries cover the core TLS 1.3 requirements of authentication, confidentiality, integrity, forward secrecy, and replay protection in the N32 roaming context
**Algorithm 2** Main Process with FS1: (* Main Process *)2: process3:     new initial_sid: session_id;4:     new initial_psk: key;5:     6:     ( (!proc_vSEPP(initial_sid, initial_psk)) | (!proc_hSEPP()) |7:     phase 1; out(c, initial_psk)8:     )
**Algorithm 3** TLS 1.3 full handshake option for N32 Roaming1:**Input:** supported_groups, certificate_chain, roaming_context2:**Output:** full handshake secure channel with forward secrecy3:**Step 1: vSEPP ClientHello Generation**4:    $client\_random\leftarrow {\left\{0,1\right\}}^{256}$5:    $x\leftarrow Z_{q}$, $client\_key\_share\leftarrow g^{x}$6:    ClientHello←(tls_1.3,client_random,client_key_share,supported_groups,certificate_ke)7:    **Event** S_STEP1_C_to_S(session_id,client_key_share,client_random,ClientHello,certificate_ke)8:    Send (ClientHello) to hSEPP9:    **Event** E_STEP1_C_to_S(session_id,client_key_share,client_random,ClientHello,certificate_ke)10:**Step 2: hSEPP ServerHello and Certificate Exchange**11:    $y\leftarrow Z_{q}$, $server\_key\_share\leftarrow g^{y}$12:    $server\_random\leftarrow {\left\{0,1\right\}}^{256}$13:    ServerHello←(tls_1.3,server_random,server_key_share,certificate_ke)14:    **Event** S_STEP2_S_to_C(session_id,server_key_share,“server_hello”,ServerHello)15:    Send (ServerHello,Certificate,CertificateRequest,ServerHelloDone) to vSEPP16:    **Event** E_STEP2_S_to_C(session_id,server_key_share,“server_hello”,ServerHello)17:**Step 3: vSEPP Certificate Verification and Key Exchange**18:    Verify server certificate chain against RAEX trust anchor19:    Validate PLMN ID in server certificate SAN records20:    $DHE\_shared\leftarrow {\left(server\_key\_share\right)}^{x}$                  ▷ Client computes shared secret21:    ES←HKDF.Extract(0,0)                               ▷ No PSK input22:    HS←HKDF.Extract(DHE_shared,derive_secret(ES,“derived”))              ▷ Includes DHE23:    $\left(tk_{c\_hs},tk_{s\_hs},tk_{c\_app},tk_{s\_app}\right)\leftarrow derive\_traffic\_keys\left(HS,transcript\right)$24:    **Event** $S\_STEP3\_C\_to\_S(session\_id,tk_{c\_app},“\mathrm{client\_finished}”,Finished)$25:    Send (Certificate,ClientKeyExchange,CertificateVerify,Finished) to hSEPP26:    **Event** $E\_STEP3\_C\_to\_S(session\_id,tk_{c\_app},“\mathrm{client\_finished}”,Finished)$27:**Step 4: hSEPP Certificate Verification and Session Completion**28:    Verify client certificate chain against RAEX trust anchor29:    Validate PLMN ID in client certificate SAN records30:    $DHE\_shared\leftarrow {\left(client\_key\_share\right)}^{y}$                  ▷ Server computes shared secret31:    Recompute handshake and application keys using DHE_shared32:    Verify client Finished message, send server Finished
**Algorithm 4** TLS 1.3 PSK-only option for N32 Roaming1:**Input:** $PSK_{shared}$, $PSK_{identity}$, roaming_context2:**Output:** PSK-only secure channel3:**Step 1: vSEPP ClientHello Generation**4:    $client\_random\leftarrow {\left\{0,1\right\}}^{256}$5:    $ClientHello\leftarrow (tls\_1.3,client\_random,PSK_{identity},psk\_ke)$6:    $ES\leftarrow HKDF.Extract(PSK_{shared},0)$7:    binder_key←derive_secret(ES,“res_binder”,ClientHello)8:    binder←HMAC(binder_key,hash(ClientHello))9:    **Event** $S\_STEP1\_C\_to\_S(PSK_{identity},PSK_{shared},client\_random,ClientHello,binder)$10:  Send (ClientHello,binder) to hSEPP11:  **Event** $E\_STEP1\_C\_to\_S(PSK_{identity},PSK_{shared},client\_random,ClientHello,binder)$12:**Step 2: hSEPP ServerHello and Key Derivation**13:    Verify binder using shared $PSK_{shared}$14:    $server\_random\leftarrow {\left\{0,1\right\}}^{256}$15:    $ServerHello\leftarrow (tls\_1.3,server\_random,PSK_{identity},psk\_ke)$16:    HS←HKDF.Extract(0,derive_secret(ES,“derived”))                 ▷ No DHE input17:    $\left(tk_{c\_hs},tk_{s\_hs},tk_{c\_app},tk_{s\_app}\right)\leftarrow derive\_traffic\_keys\left(HS,transcript\right)$18:    **Event** $S\_STEP2\_S\_to\_C(PSK_{identity},tk_{s\_app},“\mathrm{server\_finished}”,ServerHello)$19:    Send (ServerHello,Finished) to vSEPP20:    **Event** $E\_STEP2\_S\_to\_C(PSK_{identity},tk_{s\_app},“\mathrm{server\_finished}”,ServerHello)$21:**Step 3: Session Completion**22:    vSEPP verifies server Finished message23:    **Event** $S\_STEP3\_C\_to\_S(PSK_{identity},tk_{c\_app},“\mathrm{client\_finished}”,Finished)$24:    Send client Finished to hSEPP25:    **Event** $E\_STEP3\_C\_to\_S(PSK_{identity},tk_{c\_app},“\mathrm{client\_finished}”,Finished)$
**Algorithm 5** TLS 1.3 PSK-(EC)DHE option for N32 Roaming1:**Input:** $PSK_{shared}$, $PSK_{identity}$, supported_groups2:**Output:** PSK-(EC)DHE secure channel with forward secrecy3:**Step 1: vSEPP ClientHello with Key Share**4:    $client\_random\leftarrow {\left\{0,1\right\}}^{256}$5:    $x\leftarrow Z_{q}$, $client\_key\_share\leftarrow g^{x}$6:    $ClientHello\leftarrow (tls\_1.3,client\_random,client\_key\_share,PSK_{identity},psk\_dhe\_ke)$7:    $ES\leftarrow HKDF.Extract(PSK_{shared},0)$8:    binder_key←derive_secret(ES,“res_binder”,ClientHello)9:    binder←HMAC(binder_key,hash(ClientHello))10:  **Event** $S\_STEP1\_C\_to\_S(PSK_{identity},client\_key\_share,client\_random,ClientHello,binder)$11:  Send (ClientHello,binder) to hSEPP12:  **Event** $E\_STEP1\_C\_to\_S(PSK_{identity},client\_key\_share,client\_random,ClientHello,binder)$13:**Step 2: hSEPP ServerHello with DHE**14:    Verify binder using shared $PSK_{shared}$15:    $y\leftarrow Z_{q}$, $server\_key\_share\leftarrow g^{y}$16:    $DHE\_shared\leftarrow {\left(client\_key\_share\right)}^{y}$17:    $server\_random\leftarrow {\left\{0,1\right\}}^{256}$18:    $ServerHello\leftarrow (tls\_1.3,server\_random,server\_key\_share,PSK_{identity},psk\_dhe\_ke)$19:    HS←HKDF.Extract(DHE_shared,derive_secret(ES,“derived”))             ▷ Includes DHE20:    $\left(tk_{c\_hs},tk_{s\_hs},tk_{c\_app},tk_{s\_app}\right)\leftarrow derive\_traffic\_keys\left(HS,transcript\right)$21:    **Event** $S\_STEP2\_S\_to\_C(PSK_{identity},server\_key\_share,“\mathrm{server\_finished}”,ServerHello)$22:    Send (ServerHello,Finished) to vSEPP23:    **Event** $E\_STEP2\_S\_to\_C(PSK_{identity},server\_key\_share,“\mathrm{server\_finished}”,ServerHello)$24:**Step 3: vSEPP Key Computation and Session Completion**25:    $DHE\_shared\leftarrow {\left(server\_key\_share\right)}^{x}$26:    Recompute handshake and application keys using DHE_shared27:    Verify server Finished message, send client Finished28:    **Event** $S\_STEP3\_C\_to\_S(PSK_{identity},tk_{c\_app},“\mathrm{client\_finished}”,Finished)$29:    **Event** $E\_STEP3\_C\_to\_S(PSK_{identity},tk_{c\_app},“\mathrm{client\_finished}”,Finished)$
**Algorithm 6** TLS 1.3 0-RTT option for N32 Roaming1:**Input:** $PSK_{shared}$, $PSK_{identity}$, early_data_context2:**Output:** 0-RTT channel with immediate data transmission3:**Step 1: vSEPP Early Data Transmission**4:    $client\_random\leftarrow {\left\{0,1\right\}}^{256}$5:    $x\leftarrow Z_{q}$, $client\_key\_share\leftarrow g^{x}$6:    $ClientHello\leftarrow (tls\_1.3,client\_random,early\_data,client\_key\_share,PSK_{identity},psk\_dhe\_ke)$7:    $ES\leftarrow HKDF.Extract(PSK_{shared},0)$8:    client_early_traffic_secret←derive_secret(ES,“c_e_traffic”,ClientHello)9:    early_data_encrypted←AEAD.Encrypt(client_early_traffic_secret,early_data_context)10:  binder←HMAC(derive_secret(ES,“res_binder”),hash(ClientHello))11:  **Event** $S\_STEP1\_C\_to\_S(PSK_{identity},client\_early\_traffic\_secret,client\_random,ClientHello,$early_data_encrypted)12:  Send (ClientHello,binder,early_data_encrypted) to hSEPP               ▷ 0-RTT data13:  **Event** $E\_STEP1\_C\_to\_S(PSK_{identity},client\_early\_traffic\_secret,client\_random,ClientHello,$early_data_encrypted)14:**Step 2: hSEPP Early Data Processing**15:    Verify binder using shared $PSK_{shared}$16:    client_early_traffic_secret←derive_secret(ES,“c_e_traffic”,ClientHello)17:    decrypted_early_data←AEAD.Decrypt(client_early_traffic_secret,early_data_encrypted)18:    Process early data immediately                ▷ No handshake completion wait19:    $y\leftarrow Z_{q}$, $server\_key\_share\leftarrow g^{y}$20:    **Event** $S\_STEP2\_S\_to\_C(PSK_{identity},server\_key\_share,“\mathrm{early\_data\_accept}”,decrypted\_early\_data)$21:    Continue with standard PSK-(EC)DHE handshake completion22:    **Event** $E\_STEP2\_S\_to\_C(PSK_{identity},server\_key\_share,“\mathrm{early\_data\_accept}”,decrypted\_early\_data)$23:**Step 3: Handshake Completion with End of Early Data**24:    Complete DHE key exchange as in Algorithm 525:    vSEPP sends EndOfEarlyData message26:    Transition to handshake traffic keys, then application traffic keys27:    **Event** $S\_STEP3\_C\_to\_S(PSK_{identity},tk_{c\_app},“\mathrm{end\_early\_data}”,EndOfEarlyData)$28:    **Event** $E\_STEP3\_C\_to\_S(PSK_{identity},tk_{c\_app},“\mathrm{end\_early\_data}”,EndOfEarlyData)$
**Algorithm 7** TLS 1.3 0-RTT FS option for N32 Roaming (Simplified)1:**Input:** $PSK_{shared}$, $PSK_{identity}$, resumption_ticket, early_data_context2:**Output:** 0-RTT FS channel with forward secrecy and replay resistance3:**Step 1: Client Early Data with Ephemeral Keys**4:    Extract (r_SEPP_eph_pub,server_nonce,ticket_lifetime) from resumption_ticket5:    **if** current_time>ticket_lifetime **then** reject **fi**6:    Generate ephemeral key pair: (i_SEPP_eph_priv,i_SEPP_eph_pub)←(EC)DHE.KeyGen()7:    ephemeral_shared←X25519(i_SEPP_eph_priv,r_SEPP_eph_pub)8:    $early\_secret\leftarrow HKDF.Extract(PSK_{shared},ephemeral\_shared)$9:    early_key←HKDF.Expand(early_secret,“early_traffic”,transcript)10:  nonce_commitment←HMAC(early_key,server_nonce||timestamp)11:  early_data_encrypted←AEAD.Encrypt(early_key,early_data_context,nonce_commitment)12:  **Event** $S\_STEP1\_C\_to\_S(PSK_{identity},early\_key,nonce\_commitment,ClientHello,early\_data\_encrypted)$13:  Send (ClientHello,i_SEPP_eph_pub,nonce_commitment,early_data_encrypted) to hSEPP14:  **Event** $E\_STEP1\_C\_to\_S(PSK_{identity},early\_key,nonce\_commitment,ClientHello,early\_data\_encrypted)$15:**Step 2: Server Nonce Verification and Key Derivation**16:    ephemeral_shared←X25519(r_SEPP_eph_priv,i_SEPP_eph_pub)17:    $early\_secret\leftarrow HKDF.Extract(PSK_{shared},ephemeral\_shared)$18:    early_key←HKDF.Expand(early_secret,“early_traffic”,transcript)19:    **if** HMAC.Verify(early_key,server_nonce||timestamp,nonce_commitment)=false **then** reject20:    **if** replay_cache.contains(nonce_commitment) **then** reject21:    decrypted_data←AEAD.Decrypt(early_key,early_data_encrypted,nonce_commitment)22:    replay_cache.add(nonce_commitment,TTL)23:    **Event** $S\_STEP2\_S\_to\_C(PSK_{identity},early\_key,“\mathrm{verified}”,decrypted\_data)$24:    Process early_data, continue standard 1-RTT handshake completion25:    **Event** $E\_STEP2\_S\_to\_C(PSK_{identity},early\_key,“\mathrm{verified}”,decrypted\_data)$26:**Step 3: Fresh Ticket Generation**27:    Generate fresh ephemeral key pair: $\left(r\_SEPP\_eph\_priv_{new},r\_SEPP\_eph\_pub_{new}\right)\leftarrow \left(EC\right)DHE.KeyGen\left(\right)$28:    $server\_nonce_{new}\leftarrow Random\left(32\right)$29:    $ticket\_lifetime_{new}\leftarrow current\_time+TTL$30:    $new\_ticket\leftarrow CreateTicket(PSK_{new},r\_SEPP\_eph\_pub_{new},server\_nonce_{new},ticket\_lifetime_{new})$31:    **Event** $S\_STEP3\_C\_to\_S(PSK_{identity},tk_{c\_app},“\mathrm{refresh}”,new\_ticket)$32:    Send new_ticket to vSEPP33:    **Event** $E\_STEP3\_C\_to\_S(PSK_{identity},tk_{c\_app},“\mathrm{refresh}”,new\_ticket)$

#### 4.1.1. Replay Attacks on TLS 1.3 0-RTT

0-RTT replay attacks [28] exploit the zero round-trip time feature in protocols like TLS 1.3 and QUIC, where clients can send encrypted application data immediately using cryptographic material from previous connections, without waiting for server interaction. The attack works by an adversary intercepting a client’s 0-RTT messages and data, forwarding them to the server (which processes the data), but then dropping the server’s response and forcing the server to lose state (e.g., through rebooting). When the attacker replays the same 0-RTT messages, the server rejects the 0-RTT portion for security reasons but continues with a regular handshake. The client, believing the data were not delivered due to the protocol’s reliable delivery mechanism, automatically resends the same application data under the final handshake key, causing the server to process identical data twice [13]. This demonstrates that even if replay protection exists at the key exchange level, logical replays at the application level remain inevitable due to automatic retransmission mechanisms, leading TLS 1.3 to accept such replays as unavoidable rather than attempting to prevent them.

**Figure 6:**
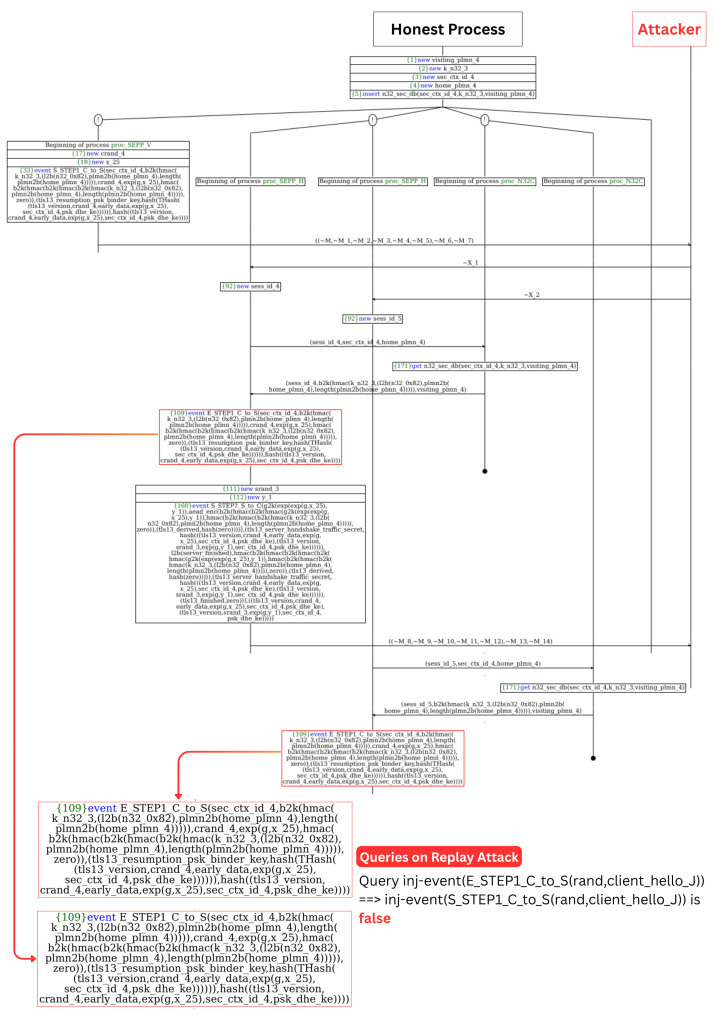
ProVerif Trace: 0-RTT replay attack, simulating N32 environment.

Figure 6 shows a diagram of the ProVerif attack trace for a typical 0-RTT replay attack scenario simulated on the N32 context. The analysis reveals a critical replay attack vulnerability where the same 0-RTT roaming message can be processed multiple times by the home SEPP. This occurs when a single SEPP-V process sends one 0-RTT message with crand_4 and x_25, but the home SEPP processes this identical message twice with different session IDs (sess_id_4, sess_id_5). Since the same cryptographic parameters are used, this results in identical early data encryption that passes validation in both instances, causing both to trigger E_STEP1_C_to_S events. This violates the injectivity property where one authentic send event corresponds to multiple receive events, enabling replay attacks on roaming signaling.

#### 4.1.2. Result

**Figure 7:**
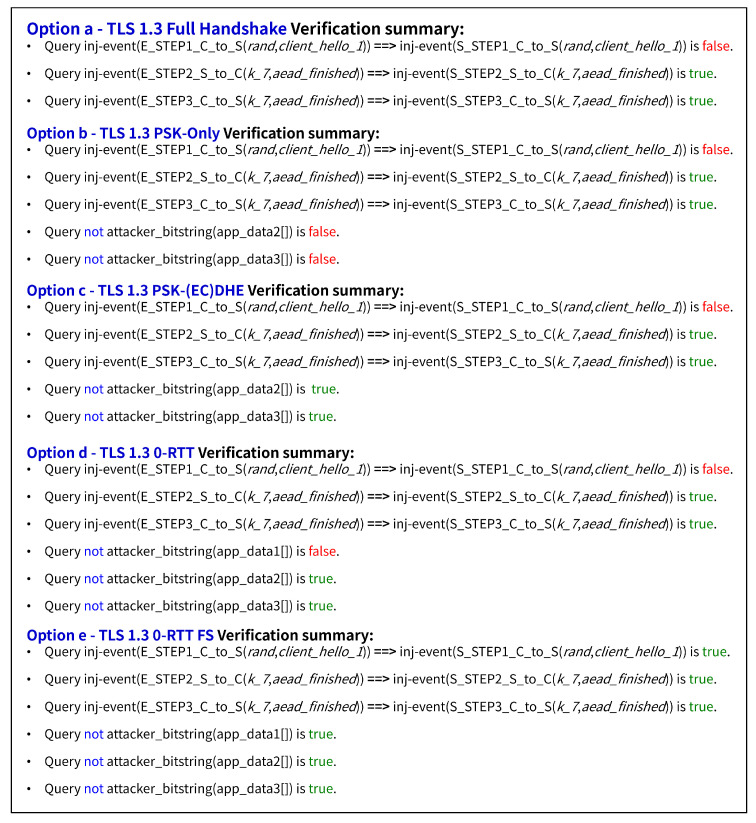
Formal verification results for TLS 1.3 options.

The ProVerif verification summary results for each TLS option are shown in Figure 7, while Table 2 provides a simple explanation of the security properties for easier interpretation. Together, these results highlight the critical security differentiators between TLS 1.3 handshake options, with particular emphasis on forward secrecy and replay attack resistance, while standard 0-RTT offers performance benefits with zero round-trips, it sacrifices forward secrecy and replay protection. The proposed 0-RTT FS solution uniquely delivers both zero round-trip performance and forward secrecy through innovative ephemeral key exchange and anti-replay mechanisms, addressing fundamental limitations in conventional TLS 1.3 implementations at the cost of moderate computational overhead. Table 3 shows a summary of the security comparison for the TLS 1.3 options.

**Critical Security Differentiators**: The formal verification of the results reveal significant differences in PFS support across options. Full handshake achieves complete PFS via (EC)DHE key exchange, ensuring that compromise of long-term keys cannot decrypt past communications. PSK-only mode lacks PFS entirely, making historical sessions vulnerable if the PSK is later compromised. PSK-(EC)DHE provides full PFS through its hybrid approach, combining PSK authentication with ephemeral key exchange. Standard 0-RTT offers limited PFS, with early data remaining vulnerable to long-term key compromise. The proposed 0-RTT FS achieves full PFS while maintaining zero round-trip performance through its innovative ephemeral key integration.

Replay attack resistance analysis reveals vulnerabilities across standard implementations. All conventional TLS 1.3 options demonstrate susceptibility to signaling-level replay attacks due to the absence of freshness mechanisms in ClientHello messages. Standard 0-RTT exhibits additional data-level replay vulnerability due to immediate early data transmission. The proposed 0-RTT FS provides comprehensive anti-replay protection through configurable defense mechanisms, including single-use ticket tracking, ephemeral key-based bloom filters, and application-level idempotency tokens.

**0-RTT FS**: The proposed 0-RTT FS option addresses fundamental limitations in standard TLS 1.3 early data mechanisms by introducing PFS while maintaining zero round-trip latency. This protocol combines PSK with fresh ephemeral DH shared secrets and implements ticket-based anti-replay protection through server-generated nonces, enabling secure early data transmission without compromising forward secrecy guarantees.

The security design of 0-RTT FS centers on three core mechanisms: forward secrecy through combined PSK and ephemeral DH key derivation using HKDF, anti-replay protection via unique server nonces embedded in resumption tickets and validated through server-side caching, and ephemeral key rotation where each ticket contains fresh server ephemeral keys. Early data encryption keys are derived from the combination of PSK, (EC)DHE shared secret (computed between client ephemeral key and server ephemeral key from ticket), and the server nonce, ensuring that compromise of long-term keys cannot decrypt past communications.

While 0-RTT FS achieves the same network round-trip count as standard 0-RTT, it incurs additional computational overhead due to (EC)DHE operations and nonce validation. This trade-off provides essential forward secrecy guarantees that standard 0-RTT lacks, making it suitable for security-critical applications where the computational cost is justified by the enhanced security properties.

**Vulnerability Assessment and Threat Model Implications**: The formal verification results expose critical limitations across standard TLS 1.3 options, particularly in replay attack resistance and forward secrecy provision. Standard 0-RTT suffers from both protocol-level and application-level replay vulnerabilities due to deterministic key derivation from static PSKs, while PSK compromise in standard implementations can expose historical session data.

Critical findings include protocol-level replay vulnerabilities in standard 0-RTT due to lack of freshness mechanisms, forward secrecy absence in PSK-based early data encryption, and significant security degradation upon PSK compromise. The 0-RTT FS option addresses these vulnerabilities through server nonce uniqueness verification and ephemeral key integration, providing replay resistance and forward secrecy at the cost of increased computational complexity. These findings demonstrate the necessity of enhanced security mechanisms for applications requiring both low latency and strong security guarantees.

## 5. Simulation Environment

### 5.1. Setup

To evaluate TLS 1.3 resumption performance for 5G cross-border roaming scenarios, we develop a comprehensive Python-based simulation framework that models N32 interface communications between SEPPs. Our simulation environment simulates the N32-c (control plane) and N32-f (data plane) protocols, with support for all standard TLS 1.3 resumption options plus our novel 0-RTT FS proposal.

The simulation architecture consists of four core components: (1) HandshakeSimulator implementing RFC 8446-compliant TLS 1.3 options with precise cryptographic timing, (2) SEPPSimulator modeling home and visited SEPP behavior with socket-based communication, (3) NetworkTopology providing realistic geographical latency simulation using NetworkX, and (4) PerformanceMonitor collecting comprehensive metrics and generating analytical reports. The system uses deterministic cryptographic operations with X25519 (EC)DHE key exchange, HKDF-SHA384 key derivation, and AES-256-GCM AEAD encryption to ensure reproducible performance measurements.

### 5.2. TLS 1.3 Options Evaluated

We evaluate five TLS 1.3 handshake options representative of different security-performance trade–offs in 5G roaming scenarios:

(a) **Full handshake**: Complete (EC)DHE with mutual certificate authentication, providing PFS and fresh authentication. Used for initial N32-c establishment.
(b) **PSK-Only**: PSK resumption without ephemeral key exchange (1-RTT), offering rapid reconnection at the cost of forward secrecy.
(c) **PSK-(EC)DHE**: Hybrid mode combining PSK with ephemeral (EC)DHE (1-RTT), maintaining forward secrecy while enabling session resumption.
(d) **0-RTT**: Zero round-trip resumption using early data keys, providing minimal latency for application data transmission.
(e) **0-RTT FS**: Our novel proposal combining 0-RTT performance with forward secrecy through server-generated ephemeral tickets and nonce-based replay protection.

### 5.3. Test Methodologies

The simulation framework supports three distinct evaluation methodologies to comprehensively assess TLS 1.3 performance across different operational scenarios:

**Simple Evaluation** performs iterative testing with configurable resumption patterns. After an initial full handshake for N32-c establishment, subsequent N32-f connections cycle through specified resumption methods. For 0-RTT FS evaluation, we pre-generate batches of 1000+ unique tickets to ensure accurate resumption timing measurement without including ticket generation overhead.

**Load Testing** simulates concurrent roaming users with configurable load patterns (1, 5, 10, 20, 50, and 100 simultaneous connections). Each user thread performs multiple N32-f exchanges using specified resumption methods, with thread-safe ticket management ensuring proper nonce uniqueness for 0-RTT FS. The results include success rates, latency distributions, and system resource utilization under varying loads.

**Geographical Testing** models realistic cross-border roaming scenarios using a mesh network topology spanning Asia (Seoul-Tokyo, 33 ms), and Americas (Los Angeles, 134 ms). Network latencies include base propagation delay, and congestion modeling using exponential distributions. Each geographical connection measures the same network conditions across all TLS options to isolate protocol performance differences.

### 5.4. Metrics and Measurements

Our performance measurement system captures fine-grained timing data across multiple operational layers. **Cryptographic metrics** include individual operation timings for key generation, (EC)DHE computation, certificate verification, and HKDF key derivation. **Protocol metrics** record round-trip counts, payload sizes, and end-to-end handshake completion times. **Network metrics** separate TLS processing time from geographical latency, enabling fair cross-regional performance comparisons.

For 0-RTT FS evaluation, we implement specialized batch processing to measure resumption performance independently of ticket generation costs. The system pre-generates cryptographically secure tickets with unique nonces, tracks their single-use consumption in a thread-safe manner, and validates replay protection mechanisms under concurrent access patterns. Table 4 shows a summary of the setup environment.

### 5.5. Performance Results

**Figure 8:**
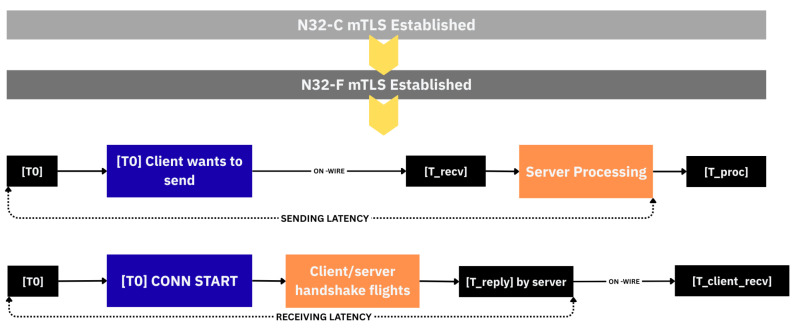
Process on time capture on N32 simulation interface.

The timing analysis of TLS 1.3 handshake performance requires careful distinction between **sending latency** and **receiving latency**, as illustrated in Figure 8. **Sending latency** represents the total time from when a protocol participant initiates transmission of a message until that message has been fully processed by the receiving party. In the context of TLS handshakes, this encompasses the computational time required to prepare cryptographic materials, perform necessary cryptographic operations such as key generation or encryption, and serialize the message for transmission. For protocols requiring handshake completion before data transmission (PSK-only, PSK-(EC)DHE, and full handshake), sending latency includes the network round-trip time as the client must wait for the server’s response before application data can be sent. Zero round-trip protocols (0-RTT and 0-RTT FS) eliminate this network dependency, allowing immediate transmission of encrypted early data alongside the initial ClientHello message. Therefore, the sending latency captures both the computational overhead of message preparation and any protocol-mandated waiting periods before secure data transmission can commence.

**Receiving latency**, conversely, measures the time from when an incoming message arrives at the receiver’s network interface until the complete response has been processed by the initiating party. This metric captures the server-side computational overhead of parsing received messages, performing cryptographic validation operations such as signature verification or decryption, generating response messages, and the network propagation time for the response to reach the client. All TLS 1.3 handshake modes incur network round-trip time in their receiving latency as the server’s response must traverse the network back to the client. The computational complexity varies significantly between protocols, with PSK-only mode requiring minimal cryptographic operations while full handshake mode demands certificate chain validation, signature generation, and ephemeral key generation. Receiving latency thus reflects both the server’s processing efficiency and the inherent network transmission delay in completing the bidirectional communication flow.

### 5.6. Latency Analysis for TLS Handshake Variants

**Figure 9:**
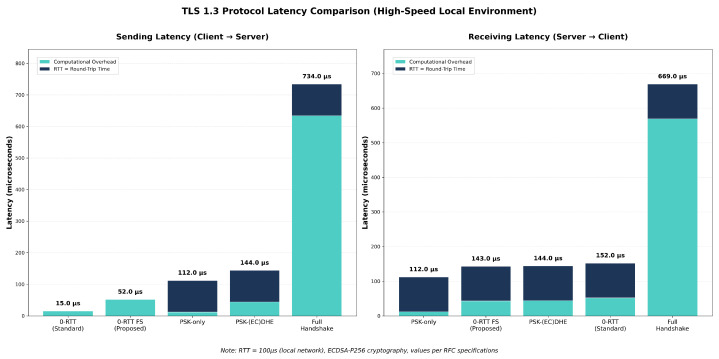
Sending latency and receiving latency per TLS option.

The simulation results presented in Figure 9 provide empirical validation of the theoretical latency predictions for various TLS handshake configurations in 5G SEPP N32 direct connections. The measurements demonstrate clear performance differentials between handshake variants across both sending and receiving phases. The total latency and summary is presented in Table 5.

#### Sending vs. Receiving Latency of TLS Options

Figure 9 compares the latency of several TLS 1.3 handshake variants in a high-speed ideal environment. Standard 0-RTT achieves extremely low client-to-server latency at 15 microseconds since early data are encrypted using only the PSK derived from the resumption ticket. However, this comes at the cost of weaker security because forward secrecy is not obtained until the server sends its fresh ephemeral key in the ServerHello. Our proposed 0-RTT FS approach shows a sending latency of 52 microseconds, which is slightly higher than standard 0-RTT due to the extra ECDH scalar multiplication performed by the client when it includes its ephemeral key in the first flight. In return, it achieves forward secrecy immediately since both client and server can derive the early traffic key using the PSK and the server’s pre-generated ephemeral key that is securely bound to the ticket during issuance.

On the receiving side, the proposed 0-RTT FS variant demonstrates a latency of 143 microseconds, which is lower than the 152 microseconds measured for standard 0-RTT and comparable to PSK-(EC)DHE at 144 microseconds. This difference arises because 0-RTT FS shifts the server’s expensive ephemeral key generation away from the critical path and into the ticket issuance stage. When resumption occurs, the server already holds its long-lived ephemeral private key and needs only a single ECDH operation to derive the forward-secret early key. Standard 0-RTT, by contrast, must complete the full ECDHE exchange during the handshake before forward secrecy is available, adding to its processing overhead. In both directions, the full handshake remains the slowest option, requiring 734 microseconds for sending and 669 microseconds for receiving due to fresh ephemeral generation and certificate validation. Overall, the results show that 0-RTT FS preserves the performance benefits of resumption while offering forward secrecy at the earliest stage of communication, improving both security and efficiency.

### 5.7. Memory Degradation Under Increasing Load

#### Memory Component Analysis

**Figure 10:**
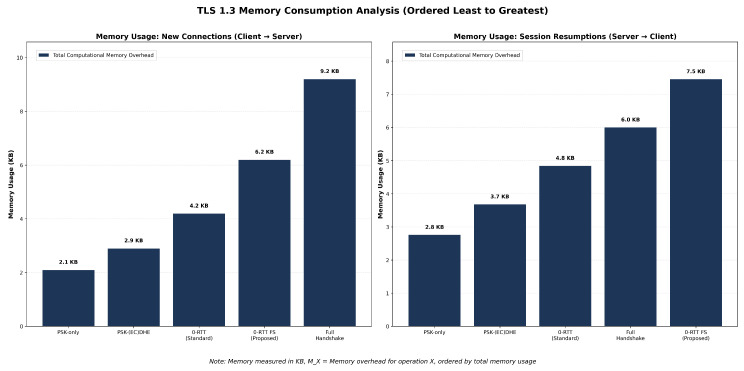
Memory component breakdown by TLS variant under different scenarios.

Figure 10 reveals the underlying drivers of memory consumption across TLS variants under two distinct deployment scenarios measured in KiloBytes (KB). In the new connections scenario (client → server), full handshake exhibits the highest memory consumption at 9.2 KB per connection, with certificate memory, ECDH operations, and signature verification contributing substantially to the memory footprint. The component analysis shows a clear progression in memory requirements: PSK-only (2.1 KB) represents the most efficient approach, followed by PSK-(EC)DHE (2.9 KB), 0-RTT Standard (4.2 KB), 0-RTT FS Proposed (6.2 KB), and full handshake (9.2 KB).

In the session resumption scenario (server → client), the memory hierarchy shifts notably, while PSK-only remains the most efficient at 2.8 KB, the 0-RTT FS Proposed variant now consumes the most memory at 7.5 KB, exceeding even the full handshake at 6.0 KB. This reversal occurs because session resumptions reduce the certificate and signature overhead in full handshakes, while the proposed 0-RTT FS variant maintains its replay protection state and forward secrecy computation requirements regardless of the connection type.

The component analysis exposes a fundamental architectural trade-off in 5G security design: the relative efficiency of different approaches depends heavily on the deployment scenario. New connection workloads show a 4.4× memory difference between the most and least efficient variants (9.2 KB vs. 2.1 KB), while session resumption workloads demonstrate a 2.7× difference (7.5 KB vs. 2.8 KB). For SEPP deployments, this analysis suggests that PSK-based approaches offer consistent efficiency advantages, while the proposed 0-RTT FS variant’s memory overhead becomes more pronounced in resumption-heavy scenarios.

### 5.8. Multi-Network Framework

**Figure 11:**
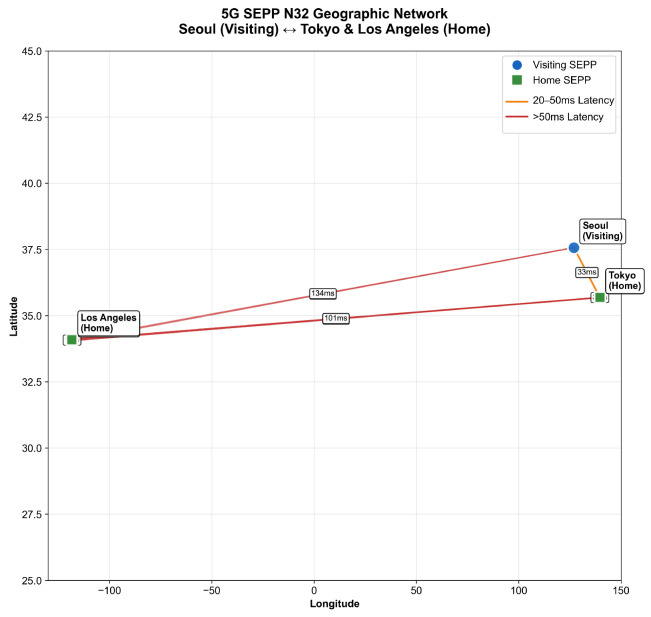
NetworkX network topology for international roaming scenario.

To evaluate TLS 1.3 performance across international roaming scenarios, we developed a comprehensive multi-network simulation framework using NetworkX for graph-based network modeling and threading for concurrent connection handling. Figure 11 shows that this simulation incorporates realistic geographical latency characteristics while providing controlled testing conditions to measure both cryptographic overhead and network topology impacts of different TLS options. The framework specifically models 5G N32 interface communications between SEPPs.

These measurements represent real-world network latencies between Seoul and major international destinations, enabling our simulation to model geographically distributed 5G roaming scenarios. The 4× latency difference between regional (Tokyo) [29] and intercontinental (Los Angeles) [30] connections demonstrates how geographic distance amplifies the performance benefits of TLS handshake optimizations. By incorporating these empirical RTT values, our mesh network model captures the realistic latency constraints faced by SEPP implementations in global roaming architectures, where the choice between security protocols becomes increasingly critical as network distance increases. This geographic modeling approach allows us to evaluate how TLS variant performance scales across different international roaming contexts, from neighboring countries with sub-50 ms connectivity to transcontinental links exceeding 100 ms round-trip times.

#### Geographical Latency Performance Analysis

**Figure 12:**
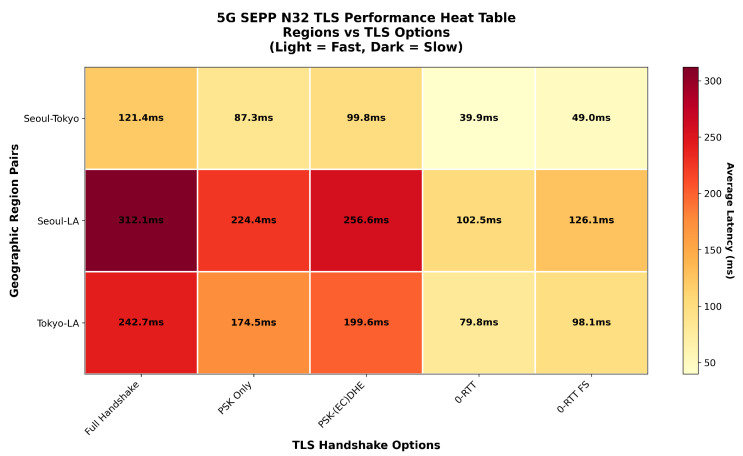
Region based latency comparison.

The graph in Figure 12 presents the latency performance of different TLS handshake variants across varying geographic region pairs in 5G roaming scenarios, with simulated latency [29,30], packet loss % and jitter. The results demonstrate the combined impact of handshake complexity and network distance on session establishment time. As expected, the full handshake consistently exhibits the highest latency across all region pairs since it requires multiple round trips and complete certificate exchange operations before application data transmission. This effect is most pronounced in the Seoul–Los Angeles path, where transcontinental propagation delay compounds the cryptographic overhead. PSK-(EC)DHE follows with substantial latency, requiring additional round trips for ephemeral key establishment despite avoiding full certificate validation.

PSK-only handshakes achieve significantly lower latency by leveraging pre-shared secrets, eliminating certificate operations while still requiring at least one network round trip for session establishment. However, performance remains constrained by network propagation delays inherent in the handshake protocol. The optimal latencies are consistently achieved by both 0-RTT variants across all geographic pairs. These protocols enable immediate application data transmission with the initial handshake flight, effectively bypassing network round-trip limitations. The proposed 0-RTT FS introduces minimal additional overhead compared to standard 0-RTT while providing forward secrecy guarantees.

Geographic distance clearly impacts absolute performance, with Seoul–Tokyo achieving the lowest latencies, Tokyo-LA showing moderate increases, and Seoul-LA demonstrating maximum latencies due to intercontinental routing. Nevertheless, the relative performance hierarchy remains consistent across all region pairs, with 0-RTT variants providing substantial advantages for latency-sensitive 5G roaming applications regardless of geographic separation between SEPPs.

## 6. Discussion

This study presents the first systematic evaluation of TLS 1.3 handshake options for 5G cross-border roaming, bridging a critical gap between cryptographic security theory and practical deployment requirements in inter-PLMN communications. By integrating formal verification with comprehensive performance analysis, our research provides mobile network operators with preliminary evidence-based guidance for understanding the security–performance trade-offs in latency-sensitive 5G roaming scenarios.

### 6.1. Principal Findings and Contributions

Our evaluation reveals a clear performance hierarchy among TLS 1.3 options based on measured handshake completion times: 0-RTT achieving 167.0 μs (baseline), our proposed 0-RTT FS at 195.0 μs (17% overhead), PSK-only at 224.0 μs (34% overhead), PSK-(EC)DHE at 288.0 μs (72% overhead), and full handshake at 1403.0 μs (740% overhead). These simulation results provide initial insights into relative performance characteristics under controlled conditions.

The 0-RTT FS proposal demonstrates competitive simulated performance while providing enhanced security guarantees. Although it introduces a 17% latency overhead compared to standard 0-RTT in our testing environment, it delivers comprehensive forward secrecy and replay protection that are absent in conventional 0-RTT implementations. This suggests that security enhancements need not necessarily impose prohibitive overhead, though real-world validation remains essential.

The formal verification results provide critical security insights, exposing a fundamental vulnerability across all standard TLS 1.3 options: susceptibility to replay attacks due to the absence of freshness mechanisms in ClientHello messages. This finding has significant theoretical implications for 5G roaming, where duplicate authentication requests could potentially compromise network integrity and billing accuracy. Our 0-RTT FS proposal addresses these vulnerabilities through cryptographically bound server nonces and ephemeral key integration.

### 6.2. Security-Performance Trade-Offs and Simulation Insights

The evaluation reveals that security–performance trade-offs in 5G roaming contexts are complex, with different protocols offering distinct advantages depending on operational requirements. PSK-only modes demonstrate favorable performance characteristics (224.0 μs) in our controlled environment, but the absence of Perfect Forward Secrecy (PFS) creates genuine long-term security considerations for international roaming partnerships.

Full handshake protocols, despite providing comprehensive security guarantees, impose substantial latency penalties (1403.0 μs) in our simulations that could potentially impact user experience in latency-sensitive applications. The PSK-(EC)DHE hybrid approach (288.0 μs) appears to offer a practical balance between security and performance based on our measurements, providing PFS while maintaining reasonable latency in the test environment.

Our 0-RTT FS proposal shows promise in simulation, achieving near-optimal performance (195.0 μs) with comprehensive security properties. However, this represents preliminary results under idealized conditions, and several deployment challenges require further investigation before practical implementation.

### 6.3. Geographical Analysis and Scalability Considerations

Our multi-network simulation incorporates latency variations from 50 ms to 371 ms across international routes, providing initial insights into how geographic distance affects protocol performance. The results suggest that optimization benefits may scale with network distance, though this relationship requires validation in production environments with diverse routing policies and quality-of-service implementations.

Load testing results suggest potential scalability advantages for resumption-based approaches under our controlled conditions. The memory efficiency analysis reveals substantial differences, with measured consumption ranging from 2.1 KB (PSK-only) to 9.2 KB (full handshake) per connection in new connection scenarios. In session resumption scenarios with increasing load, the hierarchy shifts with 0-RTT FS consuming 7.5 KB compared to full handshake’s 6.0 KB, demonstrating the computational overhead of maintaining forward secrecy and replay protection state. These variations have significant implications for large-scale deployments.

### 6.4. Deployment Considerations and Practical Challenges

The deployment of 0-RTT FS would face substantial practical obstacles that extend beyond our simulation scope. The server-side nonce tracking requirement introduces state management complexity that would need evaluation at the scale of millions of concurrent roaming sessions typical in large mobile networks. Critical factors, including network partitions, server failures, and distributed state synchronization across geographically dispersed SEPP installations, require dedicated analysis.

The mobile operator ecosystem presents unique adoption challenges compared to web-based TLS deployments. SEPP infrastructure involves multiple vendors, extensive certification processes, and risk-averse operators managing critical infrastructure. The historical precedent of TLS 1.3 adoption, which required over three years in less constrained environments [14], provides context for realistic deployment timelines. Beyond these organizational and ecosystem-level challenges, real-world network conditions themselves can also cause performance to diverge from simulation-based results, as discussed below.

#### 6.4.1. Real-World Deployment Gaps

While our simulation framework isolates cryptographic and network parameters, real-world performance may deviate due to additional factors not captured in controlled models. These include bursty or correlated packet loss, variable queuing delays, and SEPP processing bottlenecks. Prior studies show that correlated loss and jitter can significantly increase retransmission overhead in TLS sessions [31,32], while cloud-based SEPP deployments may introduce nondeterministic delays from CPU contention and virtualization overhead [33]. Such effects generally increase handshake latency and variance beyond idealized predictions, although the relative ordering of TLS modes is expected to remain stable.

To address this gap, future work should incorporate calibrated loss and jitter models or validate findings using controlled testbeds, as recommended in prior analyses of 5G latency and network modeling [34,35]. Acknowledging these deviations frames our results as preliminary but robust, highlighting the importance of field validation before operational adoption.

#### 6.4.2. Nonce Cache Scalability and Synchronization

A central element of the 0-RTT FS proposal is the server-side nonce cache, which enforces single-use tickets to prevent replay attacks, while effective in controlled settings, scaling this mechanism to carrier-grade deployments introduces additional challenges. In networks supporting millions of concurrent roaming sessions, cache lookup and insertion operations must be highly efficient to avoid becoming a performance bottleneck. Under heavy load, the latency of nonce validation could directly impact handshake completion times, especially if cache structures are not optimized for constant-time access.

Beyond local performance, geographically distributed SEPP deployments face the added requirement of maintaining replay protection across multiple sites. If nonce states are not synchronized, adversaries could exploit inconsistencies between SEPP nodes to replay early data. This problem resembles consistency trade-offs in distributed systems, where asynchronous replication or eventual consistency may introduce short windows of vulnerability. To mitigate these risks, large-scale deployments would likely require strategies such as sharded caches, Bloom filter-based membership checks, or hierarchical validation layers. Prior studies of cloud-based mobile core networks have shown that state synchronization overhead, if not carefully managed, can degrade end-to-end performance [36].

These considerations highlight that, while the nonce cache design is cryptographically sound, its real-world viability depends on engineering solutions that balance strong replay resistance with scalable, low-latency operation.

### 6.5. Preliminary Decision Framework

Based on our controlled experimental findings, we propose a preliminary decision framework to guide operators in evaluating TLS 1.3 options for inter-PLMN roaming. This framework is summarized in Table 6 and supported by the following key considerations:

- For security assessment: Formal verification reveals replay vulnerabilities in standard options, indicating potential value in enhanced approaches;
- For performance-critical scenarios: 0-RTT achieves optimal simulated latency (167.0 μs) but lacks forward secrecy; 0-RTT FS provides competitive simulated performance (195.0 μs) with comprehensive security;
- For security-sensitive deployments: PSK-(EC)DHE provides established security properties with moderate performance overhead (288.0 μs) in testing;
- For resource-constrained environments: PSK-only offers efficiency (224.0 μs) but requires security risk assessment due to absence of PFS.

These considerations are consolidated into Table 6, which presents a structured decision framework linking handshake modes, security risks, and deployment contexts.

**Table 6:** N32 TLS option preliminary decision framework for inter-PLMN roaming scenarios (simulation-based).

N32-c Initial Establishment	N32-f Protection (Reconnection)	Simulated Assessment	Risk Level	Notes
FH (mTLS)	PSK	Concerning	High	Security downgrade, no PFS
FH (mTLS)	PSK-(EC)DHE	Acceptable	Low	Maintains forward secrecy
FH (mTLS)	0-RTT	Concerning	High	Replay vulnerabilities
FH (mTLS)	0-RTT FS	Promising	Low	Requires validation
FH (mTLS)	PSK + 0-RTT	Concerning	High	Mixed mode complexity
FH (mTLS)	full handshake	Acceptable	Low	Maximum security, high latency

Following 3GPP TS 33.501, initial N32-c establishment requires full handshake with mutual TLS. Assessments based on controlled simulation results.

### 6.6. Methodological Scope and Validation Requirements

Our simulation framework provides valuable insights into relative protocol behavior under controlled conditions, though several factors limit direct applicability to production environments. The idealized network conditions do not capture the full complexity of production mobile networks, including packet loss, network congestion, and dynamic routing policies that significantly influence real-world performance.

The geographical testing incorporates realistic baseline latency measurements but cannot fully model the complexity of international routing and quality-of-service implementations. Similarly, our load testing provides initial scalability insights, though production environments involve additional complexities, including traffic patterns, connection churn, and resource contention.

Critical factors requiring dedicated analysis include energy consumption patterns, integration with existing SEPP infrastructure, vendor compatibility, and the economic incentives that drive deployment decisions in mobile operator environments.

Implementing TLS modifications in 5G environments requires more than technical validation at the operator or vendor level. Formal adoption necessitates approval from key standardization bodies, including 3GPP, which defines 5G security architecture specifications, and the IETF, which governs TLS protocol evolution. The standardization process represents an institutional prerequisite that parallels the technical validation presented in this research, as widespread deployment depends on coordinated acceptance across the telecommunications ecosystem rather than individual organizational initiatives.

### 6.7. Future Research Directions

An important next step is validating 0-RTT FS under operational roaming conditions, where network latency variations, dynamic routing policies, and scale requirements may reveal deployment challenges not apparent in controlled simulations. Equally critical is assessing the energy implications of different handshake variants on mobile devices. Recent evaluations of TLS 1.3 with advanced cryptographic mechanisms indicate that more complex operations can impose additional energy costs on constrained platforms [37]. Extending this analysis to PSK-(EC)DHE and 0-RTT FS would determine whether the enhanced security properties come with measurable impacts on mobile device battery life.

Recent work on edge computing in future wireless networks highlights the importance of distributed and hierarchical architectures for supporting low-latency, secure services in next-generation mobile environments [38]. Integrating 0-RTT FS and other handshake optimizations with edge computing frameworks could further reduce latency and improve scalability for roaming and mission-critical applications.

Additionally, adversarial testing methodologies such as those demonstrated in WAFBooster [39] could be systematically adapted to N32 signaling protocols. Automatically generated and mutated JSON/HTTP payloads would enable comprehensive assessment of SEPP resilience against malformed or intentionally evasive roaming traffic, extending beyond transport-layer security to examine application-layer vulnerabilities, including replay attacks and message tampering scenarios.

Furthermore, the emergence of automated machine learning approaches for encrypted traffic fingerprinting, such as those applied to QUIC website identification, underscores the need for robust privacy and obfuscation strategies in future protocol designs [40]. These techniques could be adapted to evaluate the resilience of 0-RTT FS and related handshake variants against traffic analysis and fingerprinting attacks in real-world deployments.

The demonstrated feasibility of combining forward secrecy with zero-round-trip operation represents a meaningful advance in cryptographic protocol design. Translating this theoretical contribution into practical deployment will require comprehensive evaluation of operational constraints and coordinated industry efforts toward standardization across the mobile ecosystem, including 3GPP and the IETF, to ensure adoption beyond research prototypes.

## 7. Conclusions

This research presents the first systematic evaluation of TLS 1.3 optimization strategies for 5G cross-border roaming, addressing critical gaps in securing inter-operator communications while maintaining ultra-low latency requirements. Through rigorous formal verification aligned with ISO/IEC 29128-1:2023 standards and comprehensive performance analysis across realistic international scenarios, we establish evidence-based guidelines for mobile operators navigating the complex security–performance landscape of N32 interface optimization. Our findings highlight the trade-offs between handshake latency, security properties, and resource overhead, providing operators with practical insights into selecting suitable TLS 1.3 options for inter-PLMN roaming scenarios.

Our performance evaluation reveals a clear hierarchy among TLS 1.3 options, with handshake completion times ranging from 167.0 μs (0-RTT) to 1403.0 μs (full handshake). The novel 0-RTT FS option achieves competitive balance at 195.0 μs, providing both PFS and replay protection while maintaining near-optimal performance. This represents only a 17% overhead compared to standard 0-RTT but delivers substantially enhanced security guarantees that are absent in conventional resumption approaches. The quantitative analysis demonstrates that PSK-only delivers efficient resumption at 224.0 μs but sacrifices forward secrecy, while PSK-(EC)DHE provides balanced security with PFS at 288.0 μs. The performance hierarchy confirms that security enhancements need not come at prohibitive costs—0-RTT FS achieves 32% performance improvement over PSK-(EC)DHE while maintaining identical security properties, challenging conventional assumptions about the security-performance trade-off.

The formal verification exposes a critical universal vulnerability: all standard TLS 1.3 options demonstrate susceptibility to replay attacks due to missing freshness mechanisms in ClientHello messages. This finding has profound implications for 5G roaming, where duplicate authentication requests could compromise network integrity and billing accuracy. Our 0-RTT FS proposal directly addresses these vulnerabilities through cryptographically bound server nonces and ephemeral key integration, making it the only option to achieve comprehensive replay resistance.

Geographical analysis reveals substantial latency variations across international routes (50 ms to 371 ms), while load testing confirms linear scalability for PSK-based options under concurrent access patterns. Memory efficiency analysis shows significant differences, with measured consumption ranging from 2.1 KB (PSK-only) to 9.2 KB (full handshake) per connection in new connection scenarios—a 4.4× variation with important scalability implications. In session resumption scenarios, the hierarchy shifts with 0-RTT FS consuming 7.5 KB compared to full handshake’s 6.0 KB, demonstrating the computational overhead of maintaining forward secrecy and replay protection state.

While our simulation-based evaluation provides valuable insights into protocol behavior and relative performance characteristics, the findings require validation in production environments to account for real-world factors, including network variability, traffic patterns, and operational constraints, that cannot be fully captured in controlled laboratory conditions. The demonstrated security vulnerabilities in standard implementations and the promising performance characteristics of our 0-RTT FS proposal warrant further investigation through field trials and industry collaboration.

As 5G networks expand globally and roaming relationships become increasingly complex, this research provides the analytical foundation necessary for optimizing secure session resumption in latency-sensitive telecommunications environments. The demonstrated feasibility of 0-RTT FS, with its sub-millisecond performance and comprehensive security properties, positions it as a compelling standardization candidate that could transform secure communications across next-generation mobile networks, ultimately contributing to improved service quality and security in international mobile connectivity.

## Figures and Tables

**Table 1:** Security requirements verification query.

Security Requirement	Verification Query
Confidentiality	Q4: attacker(App Data1)
	Q5: attacker(App Data2)
	Q6: attacker(App Data3)
Integrity	Q1: inj-event(E_STEP1_C_to_S) ==> inj-event(S_STEP1_C_to_S)
	Q2: inj-event(E_STEP2_S_to_C) ==> inj-event(S_STEP2_S_to_C)
	Q3: inj-event(E_STEP3_C_to_S) ==> inj-event(S_STEP3_C_to_S)
Mutual Authentication	Q2: inj-event(E_STEP2_S_to_C) ==> inj-event(S_STEP2_S_to_C)
	&&
	Q3: inj-event(E_STEP3_C_to_S) ==> inj-event(S_STEP3_C_to_S)
Secure Key Exchange	Q2: inj-event(E_STEP2_S_to_C) ==> inj-event(S_STEP2_S_to_C)
	&&
	Q3: inj-event(E_STEP3_C_to_S) ==> inj-event(S_STEP3_C_to_S)
PFS	Q4: attacker(App Data1)
	Q5: attacker(App Data2)
	Q6: attacker(App Data3)
Defense against replay attack	Q1: inj-event(E_STEP1_C_to_S) ==> inj-event(S_STEP1_C_to_S)
	Q2: inj-event(E_STEP2_S_to_C) ==> inj-event(S_STEP2_S_to_C)
	Q3: inj-event(E_STEP3_C_to_S) ==> inj-event(S_STEP3_C_to_S)

**Table 2:** ProVerif failing security queries for TLS 1.3 options.

Option	Failed Property and Implication
Full Handshake (a)	Q1 = false: duplicate ClientHello may be accepted, creating a signaling-level replay risk.
PSK-Only (b)	Confidentiality failure (Q4/Q6 = false): attacker can recover protected application data if PSK is compromised.
PSK-(EC)DHE (c)	Q1 = false: duplicate ClientHello may be accepted, creating a signaling-level replay risk.
0-RTT (d)	Q1 = false: replay possible at ClientHello stage.
	Q4 = false: early data confidentiality is broken, attacker can learn the first message.
0-RTT FS (e)	No failures observed: confidentiality, integrity, authentication, forward secrecy, and replay protection all hold.

**Table 3:** Security comparison of the TLS 1.3 options for N32 roaming.

Security Requirement	Full Handshake	PSK-Only	PSK-(EC)DHE	0-RTT	0-RTT FS
Confidentiality	◯	◯	◯	◯	◯
Integrity	◯	◯	◯	◯	◯
Mutual Authentication	◯	◯	◯	◯	◯
Secure Key Exchange	◯	◯	◯	◯	◯
PFS	◯	×	◯	△	◯
Defense against replay attack	×^1^	×^1^	×^1^	×^1,2^	◯

^1^ Signaling-level replay attack; ^2^ Data-level replay attack; ◯: Support; △: Limited support; ×: Not supported.

**Table 4:** Software and system environment for the N32/TLS 1.3 simulations.

Component	Details
Operating system	Ubuntu 22.04 (kernel 6.14.0-27-generic)
Programming language	Python 3.11 (CPython)
Cipher suite	TLS_AES_256_GCM_SHA256
Key exchange	(EC)DHE (Curve25519) when applicable
Authentication	X.509 mTLS (full); PSK (psk/psk-(EC)DHE); resumption ticket (0-RTT)
KDF / MAC	HKDF via HMAC-SHA256
AEAD	AES-256-GCM (12-byte nonce, seq#-XOR style)
Libraries	cryptography, psutil, stdlib (dataclasses, typing, json, time, os), networkx, asyncio
Hardware (Memory)	64GB
Host architecture	x86_64

**Table 5:** Latency performance comparison of the TLS 1.3 options for N32 roaming based on measured sending and receiving latency.

TLS Option	Sending Latency (μs)	Receiving Latency (μs)	Total Latency (μs)	Relative Performance
0-RTT (Standard)	15.0	152.0	167.0	1.00× (Baseline)
0-RTT FS (Proposed)	52.0	143.0	195.0	1.17×
PSK-only	112.0	112.0	224.0	1.34×
PSK-(EC)DHE	144.0	144.0	288.0	1.72×
full handshake	734.0	669.0	1403.0	8.40×

## Data Availability

The original contributions presented in this study are included in the article. Further inquiries can be directed to the corresponding author(s).

## Abbreviations

The following abbreviations are used in this manuscript:

x,y	(EC)DHE private keys (256-bit random values)
iSEPP,rSEPP	initiating SEPP and responding SEPP
hSEPP,vSEPP	Home Network SEPP and Visiting Network SEPP
x·G,y·G	(EC)DHE public keys on P-256 curve
SK.iSEPP,SK.rSEPP	ECDSA private keys for certificate signing
PK.iSEPP,PK.rSEPP	ECDSA public keys embedded in X.509 certificates
CERT.iSEPP,CERT.rSEPP	X.509 certificate chains with PLMN identifiers
PSK,PSK_ID	PSK and identifier from bilateral agreement
$N_{X}$	Fresh Nonce of X
PLMN_ID	Public Land Mobile Network identifier
FQDN	Fully Qualified Domain Name of SEPP endpoint
r-SEPP_eph_priv,r-SEPP_eph_pub	Server ephemeral key pair for ticket-based resumption
i-SEPP_eph_priv,i-SEPP_eph_pub	Client ephemeral key pair for 0-RTT FS
shared	Computed shared secret from X25519 ephemeral DH
ticket_id	128-bit random identifier for session resumption ticket
early_secret	Secret derived from PSK and ephemeral shared secret
early_key,early_nonce	Keys for AEAD encryption of early data
X25519(·)	Elliptic curve Diffie–Hellman function on Curve25519
ECDSA(·)	Elliptic Curve Digital Signature Algorithm
HKDF(·)	HMAC-based Key Derivation Function
HMAC(·)	Hash-based Message Authentication Code
AEAD_Encrypt(·)	Authenticated Encryption with Associated Data
Early_Secret	Initial secret derived from PSK or zero
Handshake_Secret	Intermediate secret for handshake protection
Master_Secret	Final secret for application traffic keys
NewSessionTicket	TLS 1.3 session resumption ticket message
SAN	Subject Alternative Name certificate extension
RAEX	Root Authority Exchange trust anchor
SEPP	Security Edge Protection Proxy
N32-c	N32 control interface for capability negotiation
N32-f	N32 forwarding interface for data transmission
